# COVID-19—immunity from prosecution for physicians forced to allocate scarce resources: the Italian perspective

**DOI:** 10.1186/s13054-020-03028-9

**Published:** 2020-06-05

**Authors:** Marco Ricci, Pasquale Gallina

**Affiliations:** 1 Court of Florence, Florence, Italy; 2 Department of Neurosciences, Psychology, Drug Research and Child Health, Neurosurgical Unit, University of Florence, Florence, Italy; 3 CTO Hospital, 1, Largo Piero Palagi, 50139 Florence, Italy

In times of COVID-19, it may happen that the diagnostic-therapeutic standard in a given clinical situation cannot be guaranteed [1]. This may mean denying a patient treatment because it is not available, because the only available treatment is deemed necessary for another patient, or the extreme case of having to take it away from one subject to give it to another. These decisions are made by the doctor, who in countries such as Italy, where prosecution is mandatory (Constitution art.112), will incur criminal liability.

Criminal immunity for physicians applying the necessity defense has been advanced [2]. This defense protects an individual who, in emergency situations, is forced to commit a crime to prevent more serious harm to themselves or another individual who they have a duty to protect [2, 3].

Necessity includes objectively detectable requirements: the agent is forced into the situation (e.g., the doctor who has insufficient health resources), or there is danger, not caused by the agent, of serious, existing, and inevitable damage to a subject [3] (e.g., risk of death following an established COVID-19 infection). However, necessity also establishes requisites for which subjective assessment by a judge is required. The most critical of these is the assessment of proportionality: the harm inflicted must be less than the potential harm avoided [3] (e.g., failure to administer therapy to a patient with a compromised prognosis in favor of another patient with a chance of recovery).

Physicians act according to science and conscience, supported if possible by guidelines [4]. However, guidelines have relative value and are also contrary to the principle of the equivalence of lives in liberal democracies. Therefore, a judge may not consider those guidelines as a defense thereby undermining the existence of proportionality of a doctor’s choice, thus rendering necessity inapplicable.

The legislator should recognize doctors’ actions as always proportionate, when the other conditions of necessity are documented, so that prosecution does not follow. Such modification is preferable to a generalized immunity that would risk making it impossible to prosecute offenses for negligent actions. This might be the case in Italian situations where COVID-19 patients may have been transferred to nursing homes where isolation measures were not present, causing infection in other patients hospitalized in the same facility [5].

Legal proceedings emanating from the allocation of resources during the epidemic should be handled with priority. If necessity is recognized, the costs for proceedings should be borne by the State.

## Data Availability

Not applicable.
